# *De novo* transcription of multiple Hox cluster genes takes place simultaneously in early *Xenopus tropicalis* embryos

**DOI:** 10.1242/bio.038422

**Published:** 2019-01-16

**Authors:** Mariko Kondo, Megumi Matsuo, Kento Igarashi, Yoshikazu Haramoto, Takayoshi Yamamoto, Yuuri Yasuoka, Masanori Taira

**Affiliations:** 1 Misaki Marine Biological Station, Graduate School of Science and Center for Marine Biology, The University of Tokyo, Miura, Kanagawa 238-0225, Japan; 2 Department of Biological Sciences, Graduate School of Science, The University of Tokyo, Bunkyo-ku, Tokyo 113-0033, Japan; 3 Biotechnology Research Institute for Drug Discovery (BRD), National Institute of Advanced Industrial Science and Technology (AIST), Tsukuba, Ibaraki 305-8565, Japan; 4 Marine Genomics Unit, Okinawa Institute of Science and Technology, Graduate University, Onna-son, Okinawa 904-0495, Japan

**Keywords:** RNA-seq, *Xenopus*, *De novo* transcription, Hox, Temporal collinearity

## Abstract

*hox* genes are found as clusters in the genome in most bilaterians. The order of genes in the cluster is supposed to be correlated with the site of expression along the anterior-posterior body axis and the timing of expression during development, and these correlations are called spatial and temporal collinearity, respectively. Here we studied the expression dynamics of all *hox* genes of the diploid species *Xenopus tropicalis* in four Hox clusters (A–D) by analyzing high-temporal-resolution RNA-seq databases and the results showed that temporal collinearity is not supported, which is consistent with our previous data from allotetraploid *Xenopus* *laevis*. Because the temporal collinearity hypothesis implicitly assumes the collinear order of gene activation, not mRNA accumulation, we determined for the first time the timing of when new transcripts of *hox* genes are produced, by detecting pre-spliced RNA in whole embryos with reverse transcription and quantitative PCR (RT-qPCR) for all *hoxa* genes as well as several selected *hoxb*, *hoxc* and *hoxd* genes. Our analyses showed that, coinciding with the RNA-seq results, *hoxa* genes started to be transcribed in a non-sequential order, and found that multiple genes start expression almost simultaneously or more posterior genes could be expressed earlier than anterior ones. This tendency was also found in *hoxb* and *hoxc* genes. These results suggest that temporal collinearity of *hox* genes is not held during early development of *Xenopus*.

## Abstract (precis)

**Summary:** qPCR analysis for *de novo* transcription of *hox* genes suggest that temporal collinearity is not held for all *hox* genes during early development of *Xenopus tropicalis*.

## INTRODUCTION

*hox* genes are generally considered to be involved in establishing the anterior-posterior patterning of the body axis, and are found as clusters in the genome in most bilaterians (Duboule, 2007; Durston et al., 2011; Ferrier and Minguillón, 2003). The *hox* genes in each of the clusters are designated as anterior genes (paralogous groups 1–3; PGs 1–3), central genes (PGs 4–8), and posterior genes (PGs 9–13), from the 3′ end to the 5′ end of the cluster. The order of genes in the cluster is supposed to be correlated with spatial or temporal sequential expression, and this is called collinearity. The concept of spatial collinearity has been widely accepted, and well documented in various bilaterians, in which the anterior (3′) genes are expressed anteriorly compared to posterior (5′) genes (Dekker et al., 1992; Gaunt et al., 1988; Monteiro and Ferrier, 2006). The concept of temporal collinearity has also been accepted as that the anterior genes are expressed earlier compared to more posterior genes (Durston and Zhu, 2015; Monteiro and Ferrier, 2006). The spatial order of gene expression could be considered as a result of temporal order of gene activation, and, because the coordination of gene expression is considered the main reason for the confinement of genes into a cluster; these concepts have been generally regarded as the key characteristics of the *hox* genes and the Hox cluster. However, when we scrutinized the literature, we realized that experimental evidence for the temporal collinearity hypothesis is not strong and rather incomplete, as mentioned below. Therefore, we asked whether the temporal collinearity hypothesis is applicable to all *hox* genes in a single cluster, and whether the order of *hox* gene activation follows the same rule by examining gene expression using the *Xenopus* embryos, which was one of the original animals to propose this hypothesis.

Spatial collinearity is the pattern of expression along a body axis, and has been analyzed in several model species, such as *Xenopus* (Leroy and De Robertis, 1992), mouse (Gaunt et al., 1988; Izpisúa-Belmonte et al., 1991), chick (Gaunt and Strachan, 1996), and zebrafish (Prince et al., 1998). Spatial expression of *hox* genes have been analyzed mainly using wholemount *in situ* hybridization. A link between temporal and spatial collinearity has been suggested (for example, Durston and Zhu, 2015; Gaunt and Strachan, 1996). In various review papers (such as Ferrier and Minguillón, 2003; Gaunt and Gaunt, 2016), temporal collinearity is often described in parallel with spatial collinearity and it is sometimes explained that spatial collinearity is generated by temporal collinearity, thereby leading to the impression that temporal collinearity is also generally accepted.

Compared to spatial collinearity, the experimental evidence for temporal collinearity is not strong, rather it is incomplete or ambiguous. Firstly, not all *hox* genes in a cluster have been examined so far (that is, it is incomplete). Secondly, there are variations in how temporal collinearity is interpreted; in some instances it is stated that the most 3′ gene is expressed first and more 5′ genes are expressed sequentially later (Durston et al., 2011), while some others say the physical ordering of the genes along the *hox* complexes reflects the temporal sequence of their activation (Duboule, 1992), but the definition of being expressed or activation in these contexts is ambiguous. Being expressed could be taken as ‘transcripts accumulate to a significant amount that is detectable’ which has been analyzed by RNA protection assays and *in situ* hybridization, and those results were initially and since then used as the evidence for temporal collinearity in vertebrates (Dekker et al., 1992; Dollé et al., 1989; Gaunt and Strachan, 1996; Iimura and Pourquié, 2006; Izpisúa-Belmonte et al., 1991; Wacker et al., 2004). In addition, those analyses were done with only a subset of genes in a single Hox cluster (resulting in incompleteness) or several genes from more than one hox clusters (ambiguity). As for activation, it could be taken as that ‘gene promoters are activated and new transcripts are produced in order’, but this concept has never been directly tested. If temporal collinearity means that genes (promoters, to be exact) are activated in a properly timed manner, *de novo* transcription must be analyzed for showing actual transcription. Analysis similar to detecting *de novo* transcription is to map RNA polymerase II (Pol II) binding by chromatin immunoprecipitation analysis, and this has been done in mouse tailbud tissue (Soshnikova and Duboule, 2009). Pol II was recruited roughly in a collinear manner to the region containing only posterior genes (*Hoxd10* to *Hoxd13*) in the *HoxD* cluster, with time (Soshnikova and Duboule, 2009). Since only the tailbud tissue has been used for the analysis, this partial collinearity may be a specific phenomenon that is observed in tailbud tissue, and also *de novo* transcription needs to be analyzed to confirm the order of gene activation.

The tropical (western) clawed frog, *Xenopus tropicalis*, has four Hox clusters, HoxA, B, C and D, and 38 *hox* genes are present, whereas, in the African clawed frog, *Xenopus laevis*, we found that, due to allotetraploidy, there are twice the number of Hox clusters, designated as HoxA.L, HoxA.S, HoxB.L, HoxB.S, HoxC.L, HoxC.S, HoxD.L and HoxD.S, and 76 *hox* genes, among which one is a pseudogene (*hoxb2.L*) (Kondo et al., 2017; Session et al., 2016). Therefore, there are two *X. laevis* orthologs for every *hox* gene in *X. tropicalis*, and the two genes in *X. laevis* (for example, *hoxa1.L* and *hoxa1.S*) are homeologous to each other. Previously, we have analyzed the expression of the complete set of Hox clusters and *hox* genes of *X. laevis* (Kondo et al., 2017), to overcome the above argument of incompleteness or ambiguity in analysis. We characterized developmental expression patterns of the whole set of *X. laevis hox* genes using RNA-seq which measures the amount of mature transcripts, and found that, while a subset of PG1 genes (*hoxa1.L*, *hoxb1.S* and *hoxd1.L/S*) are expressed early, there was no temporal collinearity in genes belonging to PGs 2 through 10 in any of the individual clusters, though the result with *hoxd* genes was not conclusive because of very low expression levels in many of them. This apparent lack of temporal collinearity of accumulation of mature transcripts in *X. laevis hox* genes might be a result of sub- or neofunctionalization causing differences between homeologs, or because temporal collinearity of *de novo* transcription is just hindered by looking at the accumulation of mature transcripts.

In this study, to rule out the possibility that allotetraploidization has skewed temporal collinearity of *hox* genes at the level of whole embryos, we set out to investigate the developmental expression profiles of all *hox* genes of the diploid species *X. tropicalis*, because there are no homeologs to consider. Using the precise developmental expression profiles from RNA-seq data by two groups (Collart et al., 2014; Owens et al., 2016), we first analyzed the order of accumulation of transcripts during early development. Then to analyze *de novo* transcription of *hox* genes, we performed reverse transcription and quantitative PCR (RT-qPCR) using primer sets in introns or across intron-exon boundaries for detection. The detection of *de novo* transcripts has never been reported for *hox* genes, but we successfully determined the onset of transcription of a whole set of *hoxa* genes and some of *hoxb*, *hoxc* and *hoxd* genes for comparison during development from the early blastula to late neurula stages. We found that there is no clear evidence for temporal collinearity during early development in *X. tropicalis*, neither in the accumulation of significant amounts of transcripts nor in the onset of *de novo* transcription, in whole embryos. This is the first report analyzing the onset of *de novo* transcription of *hox* genes and also with all genes in a single Hox cluster.

## RESULTS

### Accumulation of *hox* gene transcripts during early development

**Fig. 1.**
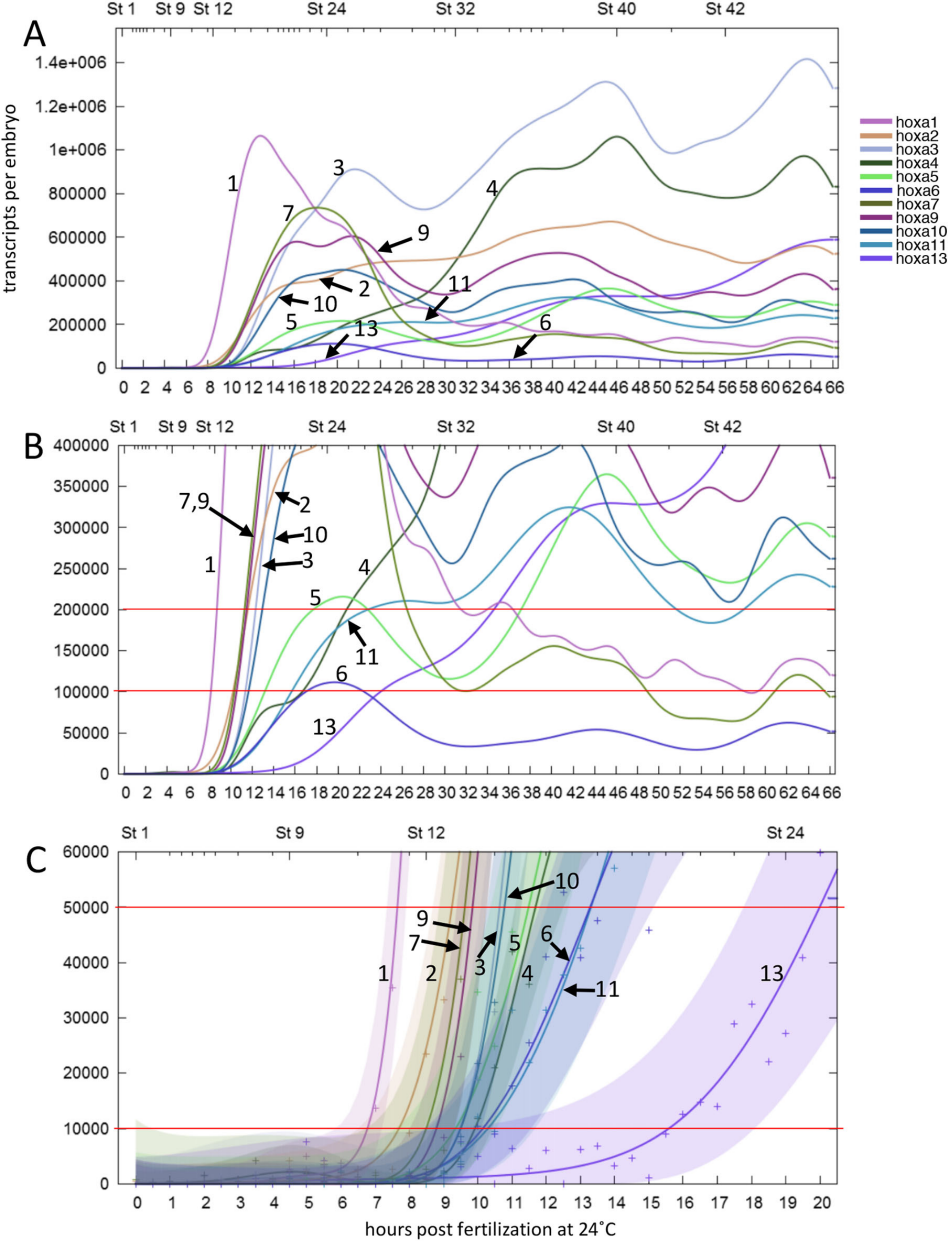
**Expression profiles of all *hoxa* genes by RNA-seq.** Graphs were retrieved from the expression profile database (http://genomics.crick.ac.uk/apps/profiles/), during the first 66 h post fertilization (hpf) (A,B) or 20 hpf (C). Numbers indicate the PG of the *hoxa* genes. Red lines are drawn at 200,000, 100,000, 50,000 and 10,000 transcripts per embryo to determine the order of accumulation (shown in Table 1). The colored regions mark Gaussian process 95% confidence intervals for each gene in C, and their overlaps show that genes reached certain numbers of transcripts simultaneously, or the precise order cannot be determined.

Based on the high-resolution expression profiles obtained by RNA-seq of *X. tropicalis* developmental stages (Owens et al., 2016), which calculated absolute numbers of transcripts in an embryo, we tried to deduce the order that *hox* genes are expressed by determining the timing that transcripts reach a certain number. Fig. 1A shows the profile of HoxA cluster genes, up to 66 h post fertilization (hpf). To compare the order of genes reaching a certain number, we arbitrarily set it to a value between 10,000 and 200,000 transcripts per embryo, to which most of the genes reach within this time period (Fig. 1B,C). Table 1 shows that the order does not change much in this range, and that, though there is a tendency that the most anterior gene *hoxa1* and *hoxa2* (PGs 1 and 2) are expressed early, and that the posterior hox genes of *hoxa11* and *hoxa13* (PGs 11 and 13) are expressed late, the order does not match the order of central genes in the cluster (PGs *3*, *4*, *5*, *6*, *7*, *9* and *10*).

**Table 1.**
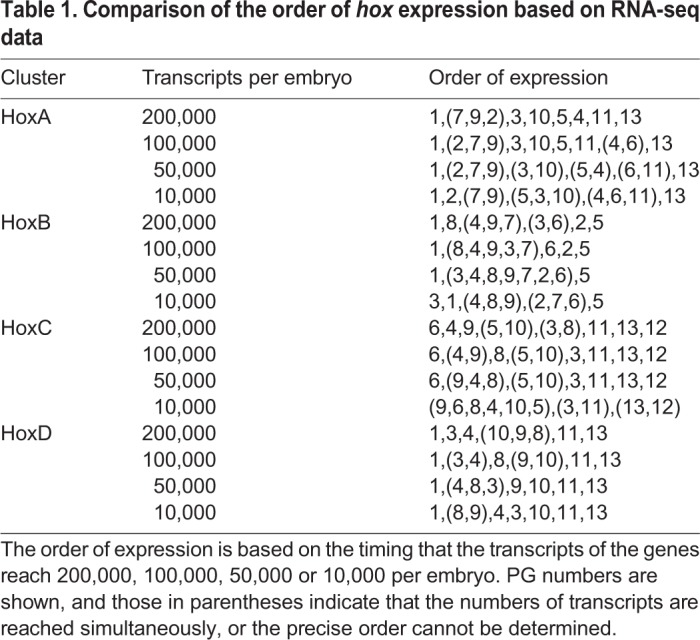
**Comparison of the order of *hox* expression based on RNA-seq data**

Similar analyses for HoxB and HoxC clusters also gave results lacking evidence for temporal collinearity, whereas HoxD gene transcripts seemed to accumulate to higher numbers in order, but several genes do not follow the order of reaching lower numbers of transcripts (Fig. S1; Table 1). Thus, the analysis using RNA-seq and observing the timing when mature transcripts accumulate to certain numbers did not totally support the temporal collinearity concept, as has been shown in *X. laevis* (Kondo et al., 2017).

### RT-qPCR method evaluation

**Fig. 2.**
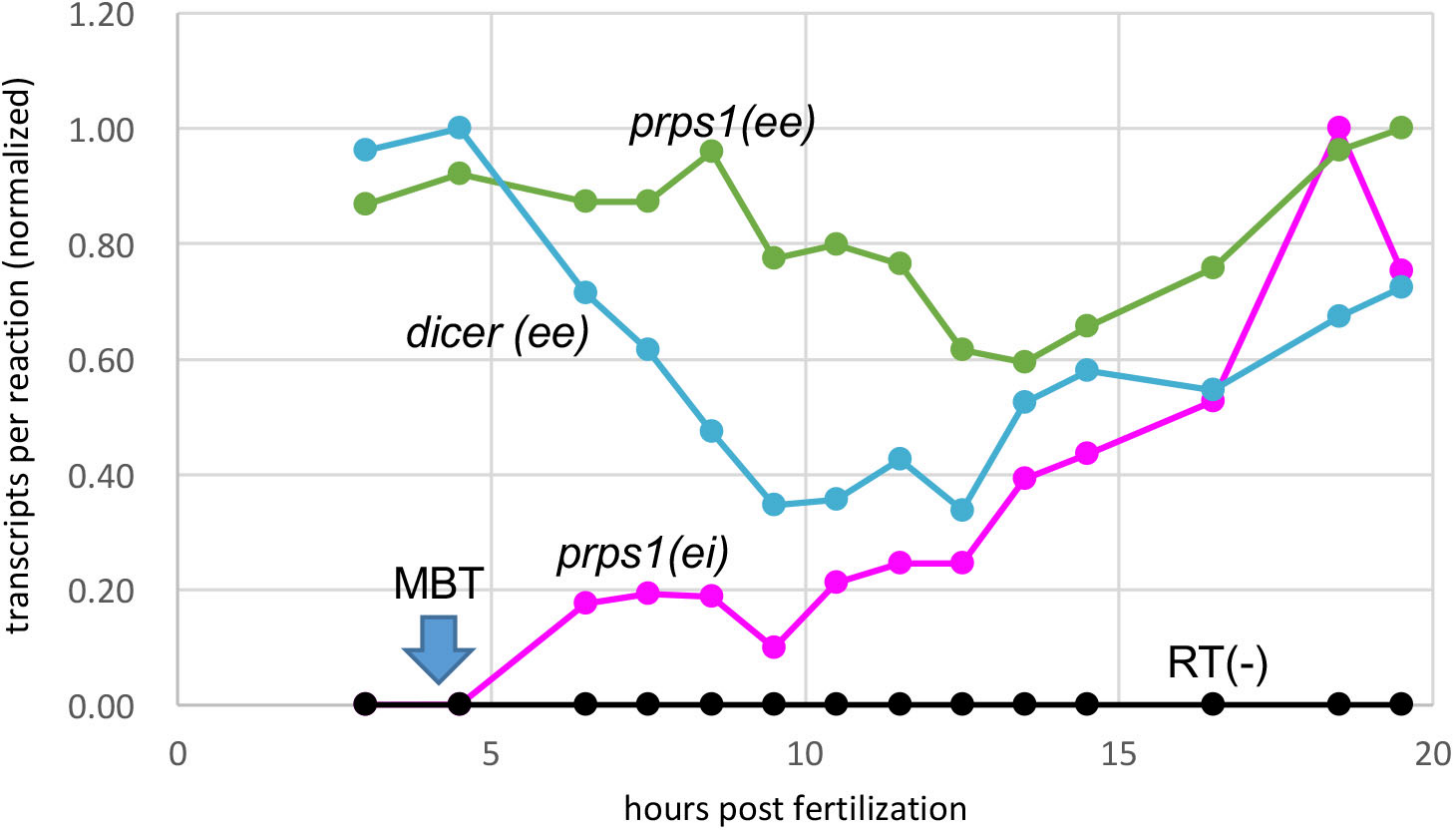
**Expression analyses of housekeeping genes *prps1* and *dicer1* by qPCR.** Primer pairs amplifying spliced transcripts (ee, mature RNA) or pre-spliced (*de novo*) transcripts (ei, precursor RNA) were used. Copy numbers per reaction were calculated and normalized taking the highest amount as 1. RT(−) are control qPCR trials for each primer set using RNA as templates (represented in black). The timing of midblastula transition (MBT) is indicated.

Because RNA-seq analyses are measurements of how much mRNA is present at a given time or in a tissue, we next examined the temporal order of gene activation in a Hox cluster, which can be estimated by detecting newly synthesized (*de novo*) transcripts. In order to measure the amount of *de novo* transcripts, we performed RT-qPCR to quantitate intronic sequences or sequences at the exon/intron boundary, i.e. pre-spliced transcripts (see the Materials and Methods). Before examining *de novo* transcription of *hox* genes, we took the housekeeping genes *prps1* (*phosphoribosyl pyrophosphate synthetase 1*) and *dicer1* and tested whether our qPCR with our RNA preparations reproduces the expression profiles from previous RNA-seq reports (Collart et al., 2014; Owens et al., 2016). Also, we tested if we could actually detect the probably low amounts of pre-spliced transcripts. We designed a primer set in an exon (‘ee’) and an intron across a splicing junction (‘ei’) for *prps1 de novo* transcripts as well as primer sets for spliced mature transcripts (for *prps1*, a set in two exons across an intron, and for *dicer1*, a set in a single exon). The amounts of transcripts, either *de novo* or mature, were measured in different developmental stages for early blastula to early tailbud stages, which are stages 7 (3 hpf) to 24 (19.5 hpf), respectively, of *X. tropicalis*. As shown in Fig. 2, mature transcripts (‘ee’ in the figure) of both genes were detected throughout the stages tested at a fairly constant level, and the expression profiles are similar to those retrieved from the database of expression profiles by RNA-seq (Fig. S2). By contrast, there was a great difference in amount of *prps1 de novo* transcripts versus mature ones, such that the *de novo* transcripts at 18.5 hpf (stage 23) was about 1/60 of the mature transcripts (Fig. S2). Furthermore, the *de novo* transcripts start to appear around the midblastula transition (MBT) and increase during development (Fig. 2). This shows that *prps1* is present as maternal transcripts, and after MBT, zygotic transcription starts and the amount of transcription per embryo increases. To check whether the amplification of intronic sequences is not due to contaminating genomic DNA into extracted RNA, all RNAs without reverse transcription (RT−) were similarly used as templates in qPCR, and virtually no amplification was detected. This verifies the validity of our experimental methods, and that we could quantitate the newly transcribed (*de novo*) transcripts despite their very low amounts. In addition, the relative consistency of expression of these two housekeeping genes between different developmental stage observed and the expression profile similar to those retrieved from the expression profile for the RNA-seq database as mentioned above indicate that taking these genes as reference, the input RNA per reaction is fairly constant, and for this reason we chose to use the same amount of RNA (or cDNA constructed from the same amount of RNA) per reaction for the following analyses.

### Quantitation of *de novo* transcripts of HoxA genes

To detect *de novo* expression of all 11 HoxA cluster genes, cDNA (RT+) and controls (RT−) from the same developmental stage were subjected to qPCR using specific primers that resided in the same intron (‘ii’) or were across an intron/exon junction (‘ei’) (Table S1). Copy numbers per reaction was deduced from each three or more assays (Fig. S3), and were compared between RT+ and RT−. We determined the timing of two aspects of activation of transcription: the start of *de novo* transcription and the onset of the active transcription, that is, when the amount of *de novo* transcripts start to increase significantly. We designated the timing as the start of *de novo* transcription when the copy number was statistically different between RT+ and RT− samples (the method we named qPCR detection). Two other methods were used to estimate the onset of active transcription: extrapolated onset and midpoint estimation, to try to avoid regarding ‘leaky expression’, if any, as active transcription. In the former method, we made linear approximations from two consecutive time points that show a significant difference in *de novo* transcript numbers between them, and deduced the intercept as the start point. The idea behind this method is that the onset of active transcription could be earlier than the last time point before the transcript significantly increased in number. The latter (midpoint estimation) was adopted since it could be hypothesized that the onset of active transcription is later than the last time point that shows no significant increase and earlier than when a significant increase in the number of transcripts is detected.

**Fig. 3.**
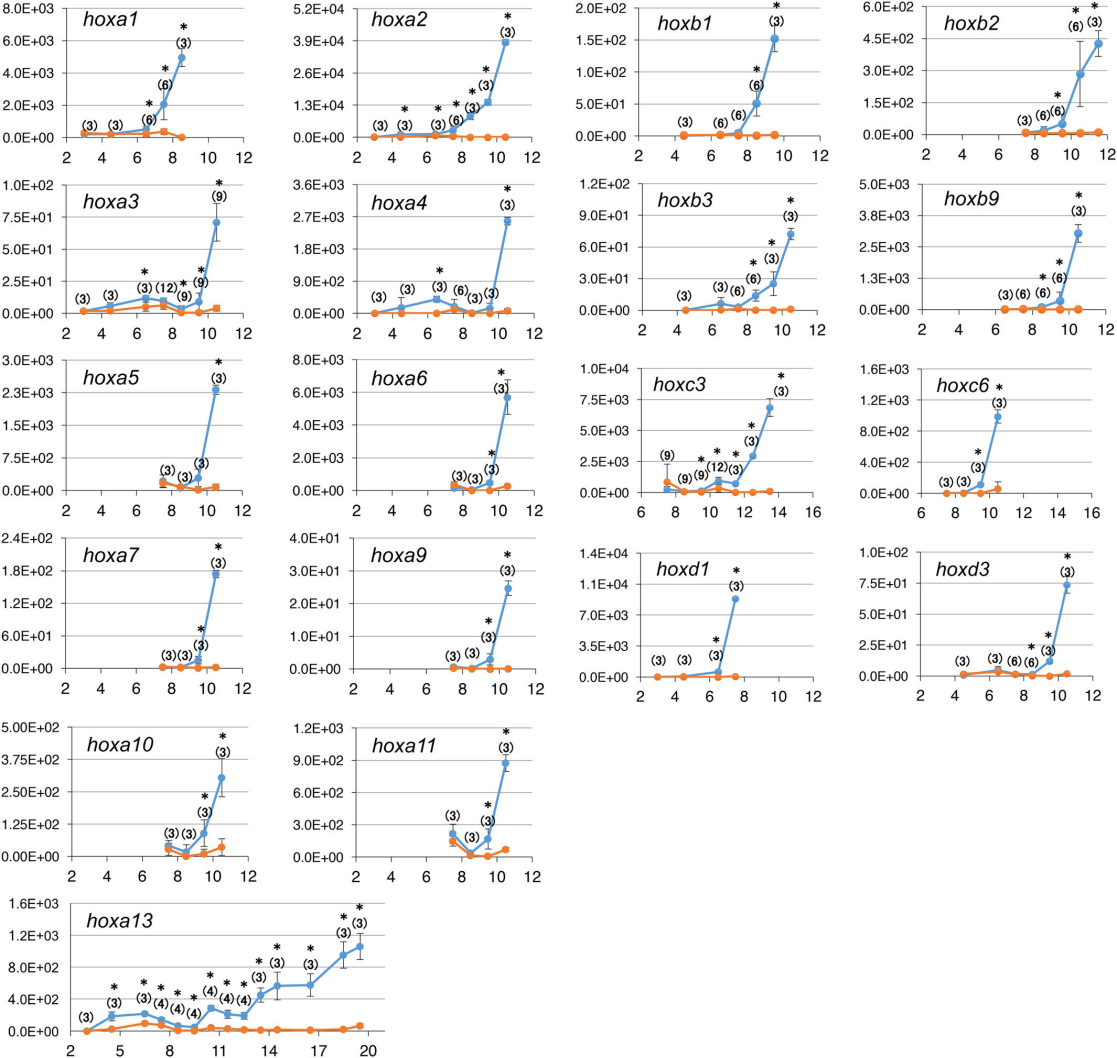
**Expression analyses of *hox* genes by qPCR.** Primer pairs amplifying pre-spliced (*de novo*) transcripts for each gene were used for qPCR. Average copy numbers per reaction and s.d. (bars) are shown for RT+ (blue) and RT− (orange) samples. The x-axis represents hpf. Numbers in parentheses are the number of trials, asterisks indicate significant difference between RT+ and RT− samples of the same stage.

As Fig. 3 shows, *de novo* transcription was detected as early as 4.5 hpf (*hoxa2* and *hoxa13*), followed by *hoxa1, 3* and *4* at 6.5 hpf, *hoxa6*, *7*, *9, 10* and *11* at 9.5 hpf, and *hoxa5* at 10.5 hpf. Because zygotic transcription mostly starts after MBT (early stage 8, around 4 hpf at 23°C), it could be thought that expression before 4.5 hpf is ‘leaky’ or premature expression, and may not be regarded as the ‘true’ onset of gene expression or active transcription. Therefore, to estimate when *hox* gene transcription starts to be fully activated, we applied the extrapolated onset method (Fig. S4; Table 2). According to this estimation, activation of transcription starts with *hoxa1* (6.3 hpf) and *hoxa2* (7.1 hpf). *hoxa10* and *hoxa13* are activated at 8.5 hpf, and then the remaining *hoxa* genes are almost simultaneously activated between 9.3 and 9.4 hpf. Similarly, the midpoint estimation method showed that *hoxa1* and *2* are activated early and *hoxa13* is late, but the other *hoxa* genes are between them and almost simultaneous. From these three methods of estimation, while the anterior genes (*hoxa1* and *2*) are early, the other genes seem to be expressed without any distinct order, and therefore the idea of temporal collinearity is not supported for HoxA cluster genes.

**Table 2.**
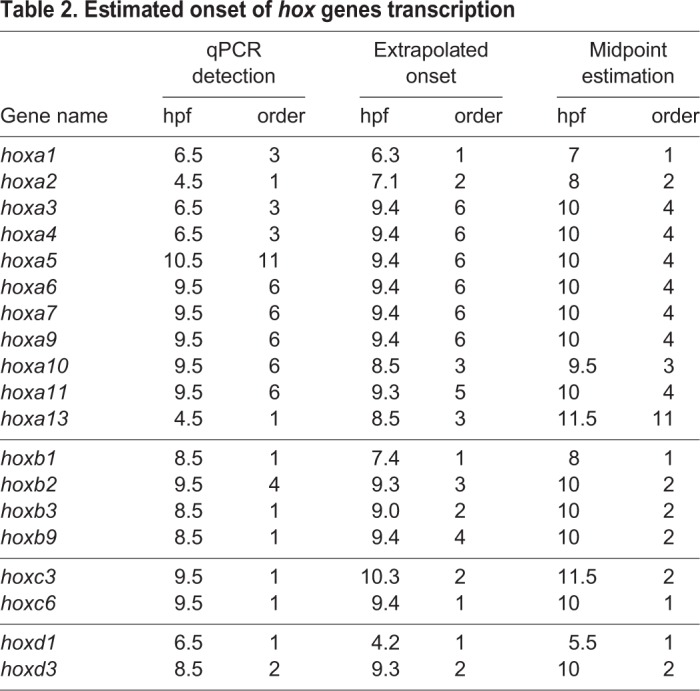
**Estimated onset of *hox* genes transcription**

### Quantitation of *de novo* transcripts of some of other Hox cluster genes

It could be possible that *hoxa* genes do not show temporal collinearity but the other clusters do, so we performed RT-qPCR analyses on those genes. From the HoxB and HoxC clusters, we selected several genes whose order of expression estimated by RNA-seq appeared to be inconsistent with the position in the cluster, namely *hoxb1*, *2*, *3* and *9*, and *hoxc3* and *6*, and also two genes from HoxD that appeared to be expressed in order according to the RNA-seq results (Fig. S1G), *hoxd1* and *hoxd3*, for comparison. As shown in Fig. 3 and Table 2, the temporal order when *de novo* transcripts are significantly detected by qPCR did not always match the gene positions of the tested *hoxb* and *hoxc* genes, again as with *hoxa* genes. By the extrapolated onset and midpoint estimation methods to estimate the onset of active transcription (Table 2), *hoxb1* was activated early, but the next anterior gene, *hoxb2* was later than *hoxb3* (extrapolated onset), or *hoxb2*, *3* and *9* could be activated simultaneously (midpoint estimation). The inversion in the order was also apparent with the two *hoxc* genes. The order of transcription for *hoxd1* and *hoxd3* was consistent with what we observed by RNA-seq analysis (Table 1).

When we combine all data from the four clusters and compare the timing of initiation of transcription, the genes belonging to the same PG do not always start transcription at the same time: PG1 genes (*hoxa1*, *hoxb1* and *hoxd1*) and PG2 genes (*hoxa2* and *hoxb2*) begin transcription at different timings, while *hoxc3* is expressed later than the other PG3 genes (*hoxa3*, *hoxb3* and *hoxd3*) which are expressed almost at the same time (see Table 2; extrapolated onset and midpoint estimation). Moreover, the order of expressed genes does not follow the order of PGs, opposed to what has been suggested for *hox* genes. Taken together, all of our data indicated that the temporal collinearity of *hox* genes is not a general rule at least during early development in *Xenopus*, when whole embryos are examined.

### Epigenetic landscape of Hox clusters during development

**Fig. 4.**
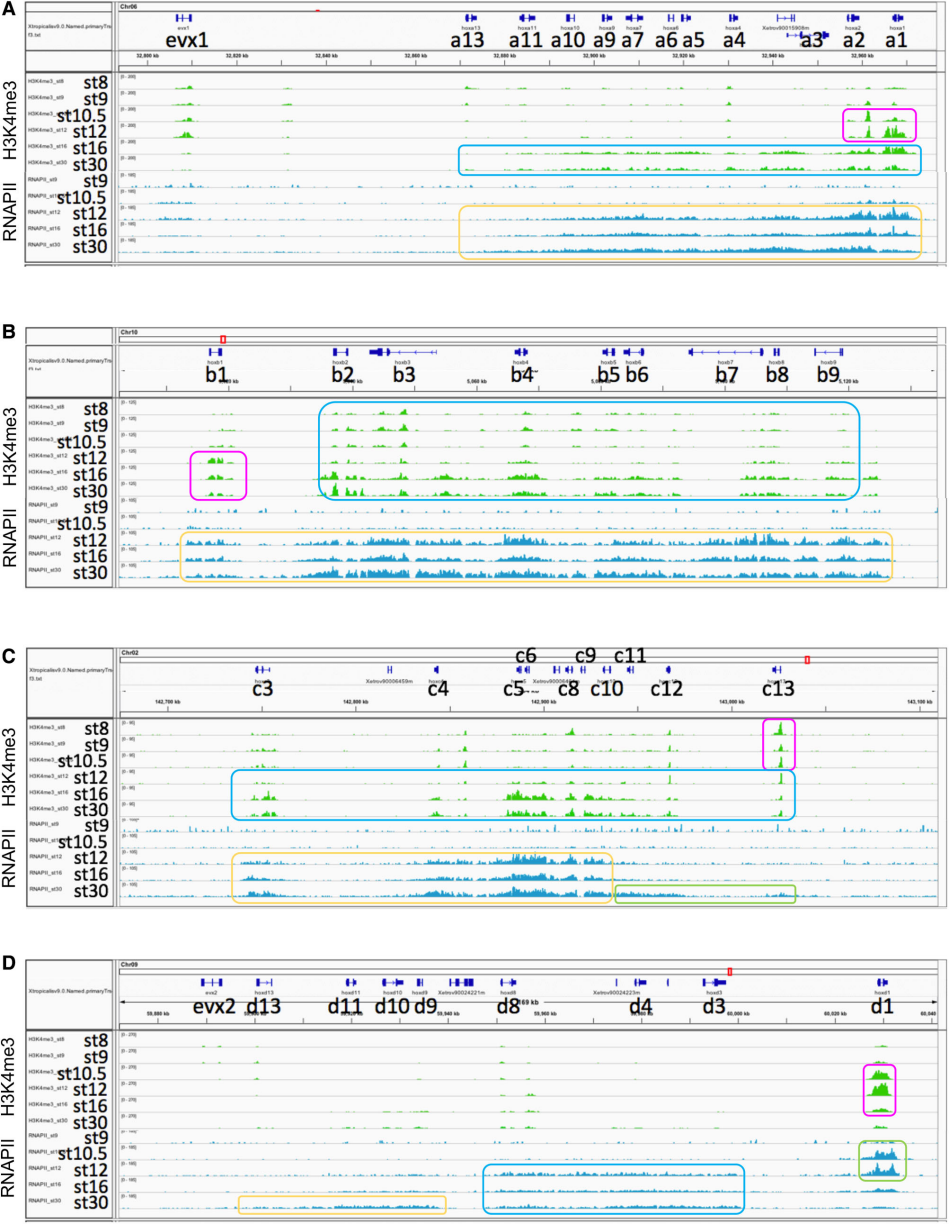
**Epigenetic and Pol II marks on the Hox** **clusters.** (A) HoxA. H3K4me3 marks were first prominent on *hoxa1* and *hoxa2* at stage 10.5 (magenta box). These marks are later at stage 16 found on a wide region of the cluster (blue). Pol II (RNAPII) marks were prominent on the entire *hoxa* region at stage12 onward (yellow), and the levels are higher on *hoxa1* and *hoxa2* than the others at stage 12. (B) HoxB. H3K4me3 marks were prominent on *hoxb2* to *hoxb9* from early stages (stages 8–10.5) onward (blue), but at stage 12, those on *hoxb1* (magenta) were relatively higher than the others, which may correspond to the qPCR results (see Table 2). RNAPII marks were apparent on all *hoxb* genes from stage 12 with no distinct order (yellow), inconsistent with the qPCR data. (C) HoxC. H3K4me3 marks were first conspicuous on *hoxc13* at early stages (magenta). The marks covered all *hoxc* genes between stage 12 and stage 16 (blue), inconsistent with the difference of initiation timing between *hoxc3* and *hoxc6*. Salient RNAPII marks were on *hoxc3* to *10* (yellow), earlier than *hoxc11* to *13* (green). RNAPII marks appeared to be relatively higher on *hoxc6* than *hoxc3* at stage 12, supporting the qPCR data. (D) HoxD. H3K4me3 marks were prominent on *hoxd1* (magenta), but almost no marks were on *hoxd3* to *hoxd13* at any stages. Increase of RNAPII binding on the HoxD cluster may be divided into three parts, *hoxd1* at stages 10.5 and 12 (green), followed by *hoxd3* to *8* (blue), then *hoxd9* to *13* (yellow), corresponding to the qPCR data of *hoxd1* and *hoxd3*. The ordinate represents mapped sequence read counts (see the Materials and Methods). Gene models for primary transcripts are shown on the top of panels.

To evaluate if epigenetic marks agree with our qPCR results, we studied the epigenetic landscape of the Hox clusters during early development, based on the published ChIP-seq data taking whole embryos (Hontelez et al., 2015). This analysis allowed us to identify active promoter marks (H3K4me3) and repressive marks (H3K27me3) on chromatin, and bodies of active genes (H3K36me3) as well as binding of the coactivator p300 (enhancers) and Pol II (RNAPII). Of them, H3K27me3, H3K36me3 and p300 marks were not so informative to discuss the order of gene activation since they did not differ between genes in the same cluster, at any given stage, for all Hox clusters (data not shown), and therefore, we focused on the active markers H3K4me3 and RNAPII (Fig. 4).

On the HoxA cluster, the active promoter mark H3K4me3 for *hoxa1* and *hoxa2* appeared to increase from stage 9 onward, coinciding with the observation that these two genes start to be transcribed rather early in this cluster (see Table 2). Levels of H3K4me3 became higher in *hoxa3* to *hoxa10* at stage 16 and stage 30, and there seemed to be no significant difference in timing among these genes. *hoxa11* and *hoxa13* appeared to be kept at a low-activated state. RNAPII, indicating active or poised transcription, widely occupies *hoxa1* to *hoxa10* genes by stage 16 without any temporal differences between genes, whereas RNAPII occupancy to *hoxa11* and *hoxa13* appears to occur slightly later. Thus, almost simultaneous initiation of transcription of *hoxa3* to *hoxa10* around 9 or 10 hpf (around stage 12) seems to coincide with developmental changes of the active marks of H3K4me3 and RNAPII, supporting the qPCR results. However, on Hox B, C and D cluster genes, the levels of H3K4me3 are consistent in some cases but not in others with the order of *de novo* transcription (see Fig. 4), and RNAPII occupancy corresponded to the qPCR data of *hoxc* and *hoxd*, but not well *hoxb* genes examined. Taken together, we concluded that epigenetic marks H3K4me3, H3K27me3, H3K36me3 and p300 may not be major regulators common to all Hox clusters for determining the order of gene activation.

## DISCUSSION

In our previous study (Kondo et al., 2017), we suspected that the concept of temporal collinearity does not hold true in the allotetraploid frog *X. laevis*, when whole embryos are taken and subjected to RNA-seq analysis, and the patterns of expression were used to evaluate the timing of expression. In the current analysis, we tested this concept using the diploid frog *X. tropicalis*. We analyzed all the *hox* genes in *X. tropicalis* using the RNA-seq data (Owens et al., 2016) to determine the timing that accumulated transcripts reach a certain level, and in selected *hox* genes including all *hoxa* genes, the timing when *de novo* transcription begins or becomes activated, by RT-qPCR for mRNA precursors. Our results suggest that the temporal collinear pattern of expression and epigenetic marks during early development does not exist in the genes of the HoxA, HoxB and HoxC clusters.

It has been postulated that clustering of *hox* genes may be prerequisite for temporal collinearity to be established. This assertion is based on two assumptions: (i) temporal collinearity exists in vertebrates; and (ii) in invertebrate species whose Hox cluster is disrupted, temporal collinearity is not observed (Monteiro and Ferrier, 2006). In addition, the possible interlock between temporal and spatial collinearity of *hox* genes as mentioned above may be another reason for widely accepting the temporal collinearity hypothesis as a dogma. So, when the most anterior *hox* gene (PG1) is detected early and the most posterior *hox* genes (e.g. PGs 11–13) are detected late, this has been accepted as the evidence for temporal collinearity, even though no other clear collinearity in between (PGs 2–10) is observed, as exemplified in Pascual-Anaya et al. (2018). It may be argued that such partial disorder of apparent collinearity should be acknowledged as temporal collinearity being present, because temporal collinearity of gene activation could be hidden or ambiguous owing to just analyzing the amount of accumulating mRNA (that is, by RNA-seq analysis).

So, to settle this argument, we quantitated *de novo* transcription in this paper. By analyzing all the genes from the HoxA cluster by RT-qPCR, we demonstrated that the *hoxa* genes do not start *de novo* transcription one after another in order corresponding to the position in the cluster, though *de novo* transcripts are detected earlier for anterior genes, but the most posterior genes are not always later than others. In fact, there is no apparent order of transcription and many genes start to be transcribed almost simultaneously. As a result, the orders of *de novo* transcription (gene activation) were more ambiguous in terms of collinearity, compared to those of mRNA accumulation. In addition, we found that transcription of posterior genes begins quite early. To generalize the data from HoxA, we further showed that the same trend was observed for *hoxb* and *hoxc* genes. In conclusion, we could not obtain any evidence to support the temporal collinearity hypothesis at least from HoxA, B and C, in the activation of transcription as well as in the accumulation of mRNA.

A criticism to this conclusion might be that the *de novo* transcription of *hox* genes should be quantitatively analyzed with separate germ layers or single cells. This could be reasonable if such analysis had been conducted for producing evidence supporting temporal collinearity. Recently, single-cell transcriptome analysis of *X. tropicalis* embryos has been performed (Briggs et al., 2018) and precise analysis may elucidate the order of accumulation of *hox* genes in each germ layer or tissue. Nevertheless, the order of *de novo* transcription cannot be determined from single-cell transcriptomes. Assuming if we are able to detect *de novo* transcription of any gene by RT-qPCR and if temporal collinearity holds in all germ layers, theoretically, the order of *de novo* transcription with whole embryos will always reflect that in an individual germ layer, and the order will never be reversed even if the onset of transcription is different between germ layers (Fig. S5), as far as the initial level of *de novo* transcripts is detectable (that is, above the detection limit indicated by dashed horizontal lines in Fig. S5). Thereby, our results showing reversals in the order of expression suggest that temporal collinearity does not hold in all tissues or germ layers in the embryo.

We reached the same conclusion in the epigenetic landscape of any Hox cluster, in which neither active marks increase nor repressive marks decrease sequentially in the order according to the position of the gene in the Hox cluster (Fig. 4), against the expectation that this is the case if temporal collinearity exists. The epigenetic landscape did not always coincide with the qPCR results and the increase in activation marks, especially RNAPII occupancy, were observed later than the deduced onset of transcription. We speculate that it is because our qPCR detection is much more sensitive. Inductive regulation of *hox* genes may enable us to explain the pattern of expression, accumulation or *de novo* transcription, observed in our study. That is, it is reported that the most anterior *hoxa1*, *hoxb1* and *hoxd1* (Kolm and Sive, 1995; Kudoh et al., 2002; Papalopulu et al., 1991; Sive and Cheng, 1991), and some other *hoxb* genes (Dekker et al., 1992) are induced by retinoic acid, and central *hoxb* genes (*hoxb6*, *hoxb7* and *hoxb8*) are induced by FGF (Bel-Vialar et al., 2002).

Temporal collinearity has been considered a major characteristic of *hox* genes and Hox clusters. However, our results suggest that temporal collinearity in early development may not be the reason why *hox* genes are confined in clusters. Instead, it may be more likely that the presence of genes as clusters is not directly linked to temporal collinearity in transcription. In any case, it should be noted that, to begin with, temporal collinearity itself has never been demonstrated at the level of *de novo* transcription.

Our analysis on *de novo* transcription of *hox* genes opens the door to further investigating how Hox clusters and *hox* genes are activated, not only during early development, but in tissue differentiation such as in tail or limb formation. As mentioned earlier, we previously showed from RNA-seq of *X. laevis hox* genes that temporal collinearity does not hold for individual clusters and that the timing of transcription of homeologous L and S genes are not always the same (Kondo et al., 2017). Therefore, further analysis of *de novo* transcription of the *X. laevis* homeologous genes as well as a whole set of *hoxd* genes of *X. tropicalis*, which relatively shows temporal collinear accumulation of mRNA, will reveal whether or not all genes are transcribed in the order in the clusters, which is a very interesting and inevitable subject to work on the temporal collinearity hypothesis.

## MATERIALS AND METHODS

### Analysis of RNA-seq data of *X. tropicalis*

Expression patterns of *X. tropicalis* genes based on RNA-seq data by two groups (Collart et al., 2014; Owens et al., 2016) are available online at Searchable Database of *X. tropicalis* Gene Expression Profiles (http://genomics.crick.ac.uk/apps/profiles/). We chose the dataset from Owens et al. for our analysis because it is the most comprehensive and high-temporal resolution one for normal *X. tropicalis* embryos at present. The ClutchA polyA+ dataset from Owens et al. was used for the analyses, but the results did not largely differ when the other datasets (Clutch A rdRNA or Clutch B polyA+) were used. To estimate the order of expression, we drew lines at 200,000, 100,000, 50,000 or 10,000 transcripts, and deduced the order of genes that the transcripts reached these numbers. According to Owens et al. (2016), the expression data are shown as curves with Gaussian process 95% confidence intervals, and when we take this confidence intervals into consideration, the order of expression for some genes (or in some cases, most genes) could not be determined. Therefore, when the timing did not differ much (within about 30 min from each other), we considered them to be indifferent (simultaneous).

### Collection of *X. tropicalis* embryos and extraction of total RNA

*Xenopus tropicalis* was provided by Amphibian Research Center (Hiroshima University, Japan) through AMED under Grant Number JP18km0210085. *Xenopus* *tropicalis* embryos were obtained by artificial insemination and cultured in 10% Steinberg's solution at 23°C and were sampled at 3 (stage 7), 4.5 (stage 9), 6.5 (stage 10), 7.5 (stage 10∼10.5), 8.5 (stage 11∼11.5), 9.5 (stage 12), 10.5 (stage 12.5), 11.5 (stage 13), 12.5 (stage 15), 13.5 (stage 17), 14.5 (stage 18), 16.5 (stage 21), 18.5 (stage 23) and 19.5 (stage 24) hpf. Ten embryos each were collected into ISOGEN (Nippon Gene) and homogenized using a plastic pestle, and stored at −80°C until extraction. Total RNA was extracted by standard methods. The amount of total RNA was not different among different embryonic stages, as reported previously by Sagata et al. (1980).

### RT-qPCR

Two micrograms of total RNA was reverse-transcribed (RT+ samples) using SuperScript III (Invitrogen) with random pentadecamers (N_15_ primers) (Stangegaard et al., 2006) in a 20 µl reaction. The final concentration of N_15_ primers in the reverse-transcription reaction mix was 25 µM, and this concentration was determined so that the amplification efficiency reached the maximum. To use as control, the same RNA was incubated in the same reaction mix in the absence of reverse transcriptase (RT− samples). *De novo* expression of genes was estimated by detection of pre-spliced mRNA. Primers for qPCR were designed so that sequences in the exon (‘ee’ primer set), the intron (‘ii’ primer set) or around a splice junction (‘ei’ primer set) were amplified (Table S1), including those for housekeeping genes *dicer1* (NCBI Acc. No. NM_001129918) and *prps1* (NM_203809). PCR products were cloned into pGEM-T easy vector (Invitrogen). Either the plasmid DNA or its insert which was amplified by PCR using primers in the vector sequence and gel-extracted to remove primers, was quantified and used for calibration. qPCR was performed using SYBR Premix Ex TaqII (TaKaRa) and Light Cycler (Roche), according to the manufacturers' protocols. cDNA from 5 ng RNA was used in 10 µl reactions, except for those for *hoxa11*, in which twice as much cDNA was used. The annealing temperature was either 55 or 60 degrees (Table S1). RT- preparations were subjected to qPCR as controls to check contaminating genomic DNA in the extracted RNA. Melting curves were checked for every amplification product to assess the validity of the amplification. A standard dilution curve for each primer–probe combination was drawn for quantification of the transcripts. The amounts of transcripts were deduced only from qPCR data that fit this dilution curve in the range between the lowest and highest amount of template with which the dilution curve was drawn. Each cDNA sample was analyzed by qPCR in triplicate, and the estimated copy number values for RT+ and RT− samples were compared (*F*-test, then *t*-test). The onset of *de novo* expression was determined by three methods. The first (qPCR detection) was to take the earliest time point when a significant difference was detected between RT+ and RT− samples. Alternatively, the values of RT− were subtracted from those of RT+, and the difference in the number of transcripts at each time point was analyzed by the Tukey–Kramer multiple comparison. Values of *P*<0.05 were considered statistically significant. As the second method (extrapolated onset), the earliest two consecutive time points that showed a significant difference in the number of transcripts between each other were identified. A linear line connecting these two coordinates was drawn and its intercept with the x-axis (time) was deduced as the onset of *de novo* transcription, except for *hoxc3*. Since *hoxc3* showed a gradual increase in transcript numbers between 10.5 hpf and 12.5 hpf (no difference was detected between 10.5 and 11.5 hpf or 11.5 hpf and 12.5 hpf, but a significant difference between 10.5 and 12.5 hpf), a linear regression line was drawn from these three points. As the third method (midpoint estimation), the midpoint of the above two consecutive time points was determined as the index for active transcription, again except for *hoxc3*. For *hoxc3*, we determined the midpoint to be 11.5 hpf, between 10.5 and 12.5 hpf (Table 2).

### Epigenetic analysis

ChIP-seq data of epigenetic marks in *X. tropicalis* embryos (Hontelez et al., 2015) were represented by using IGV genome browser and *X. tropicalis* genome and gene models v9.0. ChIP-seq reads were mapped with bowtie2 and tgf files were generated with ‘Count’ command of igvtools (window size, 25; extension factor, 120). Promoter histone marks (H3K4me3), repressive histone marks (H3K27me3), actively transcribed regions (H3K36me3), enhancer marks (p300) and enrichment of RNA polymerase II (RNAPII) were indicated with gene models for Hox clusters.

## Supplementary Material

Supplementary information
