# Supplementary material for: De novo transcription of multiple Hox cluster genes takes place simultaneously in early Xenopus tropicalis embryos
Source: Biol Open. 2019 Jan 16;8(3):bio038422. doi: 10.1242/bio.038422 (PMC6451350; doi:10.1242/bio.038422)
Supplement: Supplementary information [file biolopen-8-038422-s1.pdf]

Fig. S1 (Kondo et al.)

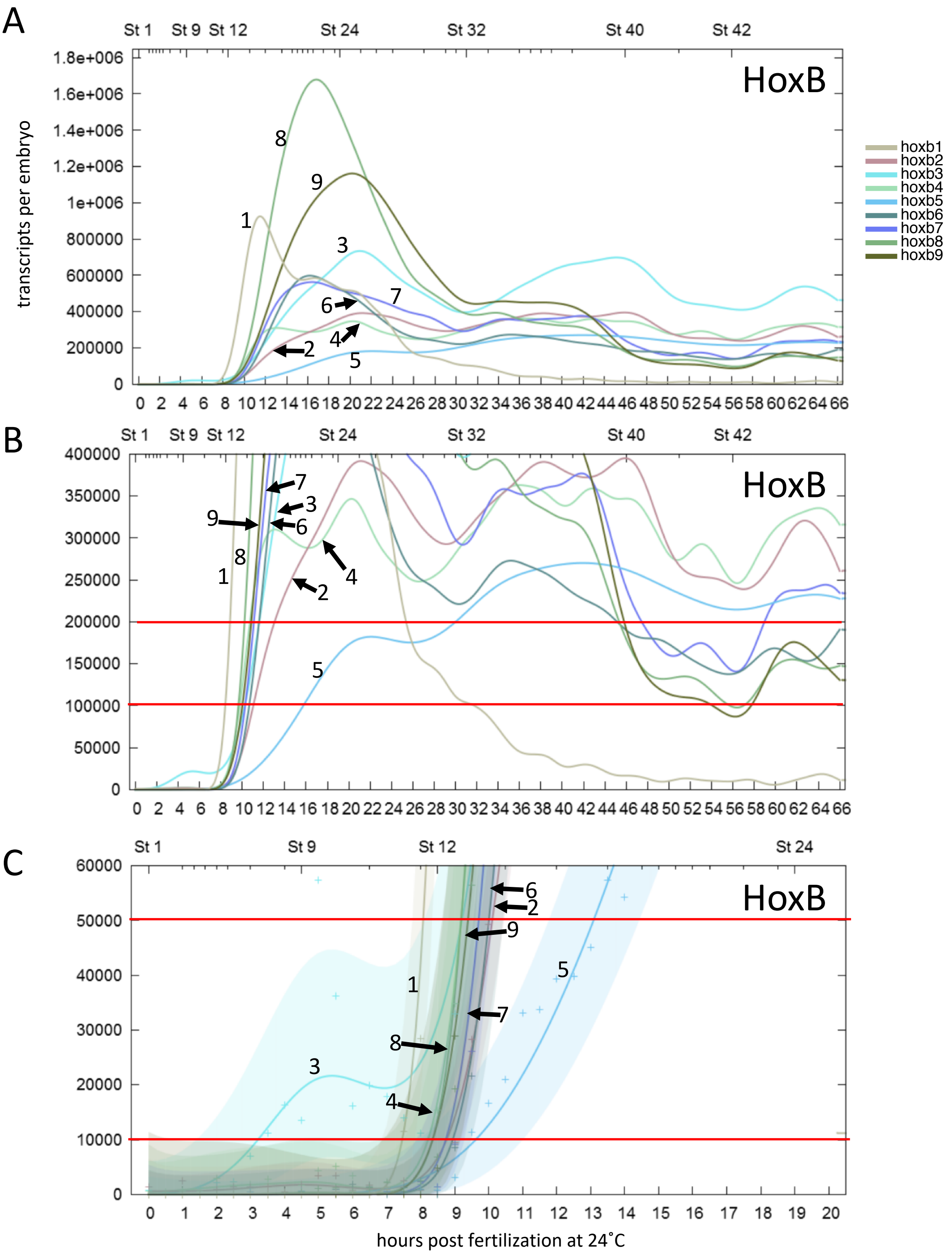

Fig. S1. Expression profiles of *hoxb*, *hoxc*, and *hoxd* genes by RNA-seq.
Expression profiles of all *hoxb* (A-C), *hoxc* (D-F), and *hoxd* (G-I) genes were retrieved from the expression profile database (http://genomics.crick.ac.uk/apps/profiles/). PG numbers are indicated. Red lines are drawn at 200,000, 100,000, 50,000, and 10,000 transcripts per embryo (the same as shown in Table 1). The colored regions mark Gaussian process 95% confidence intervals for each gene in C, F and I, and their overlaps show that genes reached certain numbers of transcripts simultaneously, or the precise order cannot be determined.

Biology Open • Supplementary information

Fig. S1 (continued) (Kondo et al.)

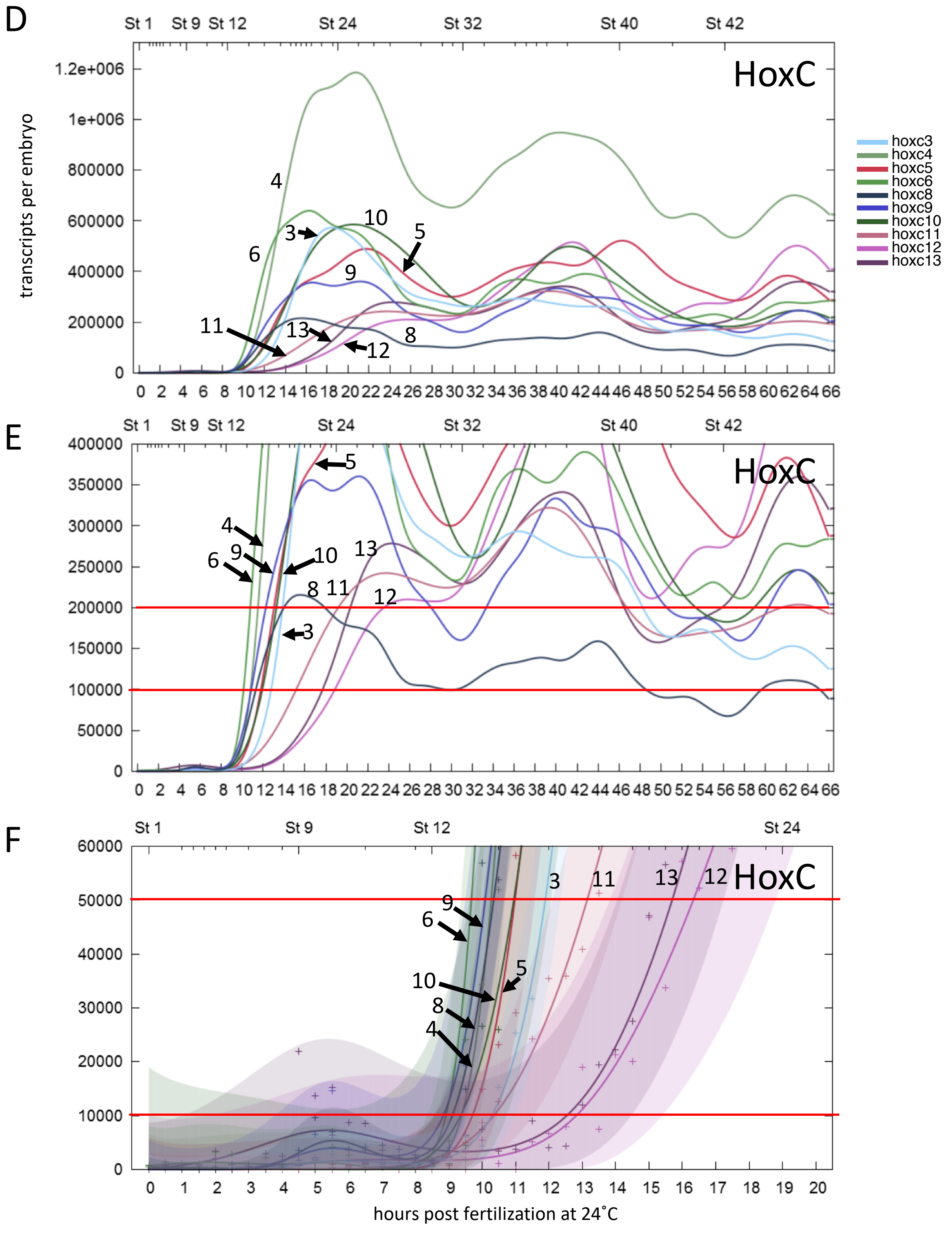

Biology Open • Supplementary information

Fig. S1 (continued) (Kondo et al.)

G

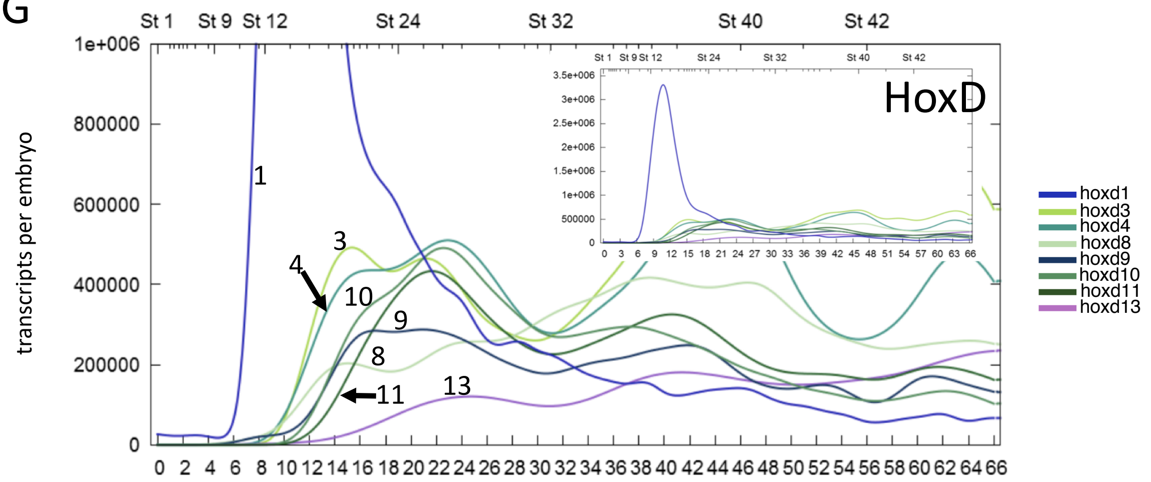

H

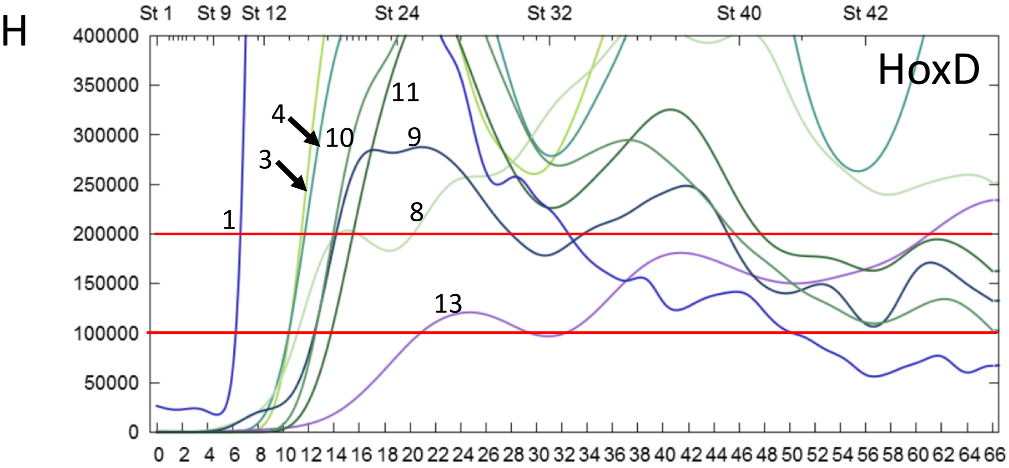

I

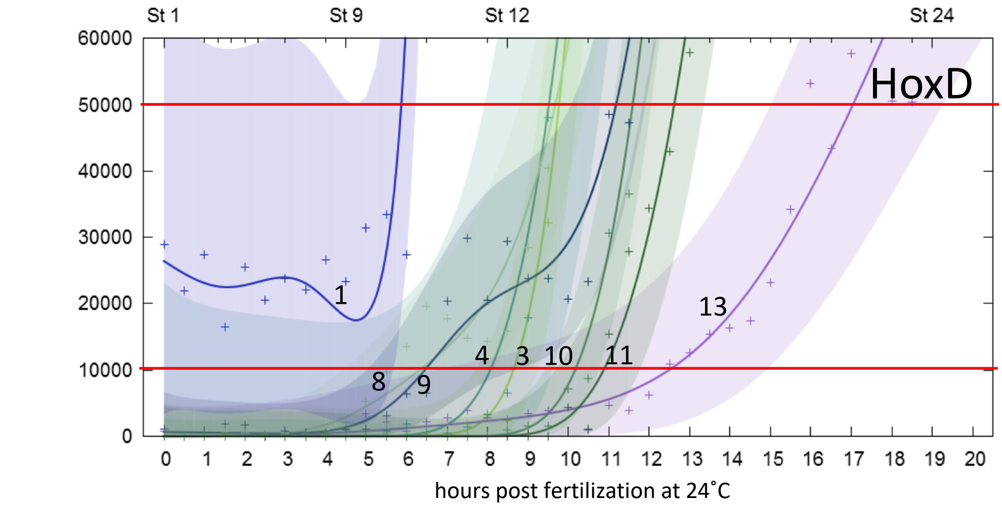

Biology Open • Supplementary information

Fig. S2 (Kondo et al.)

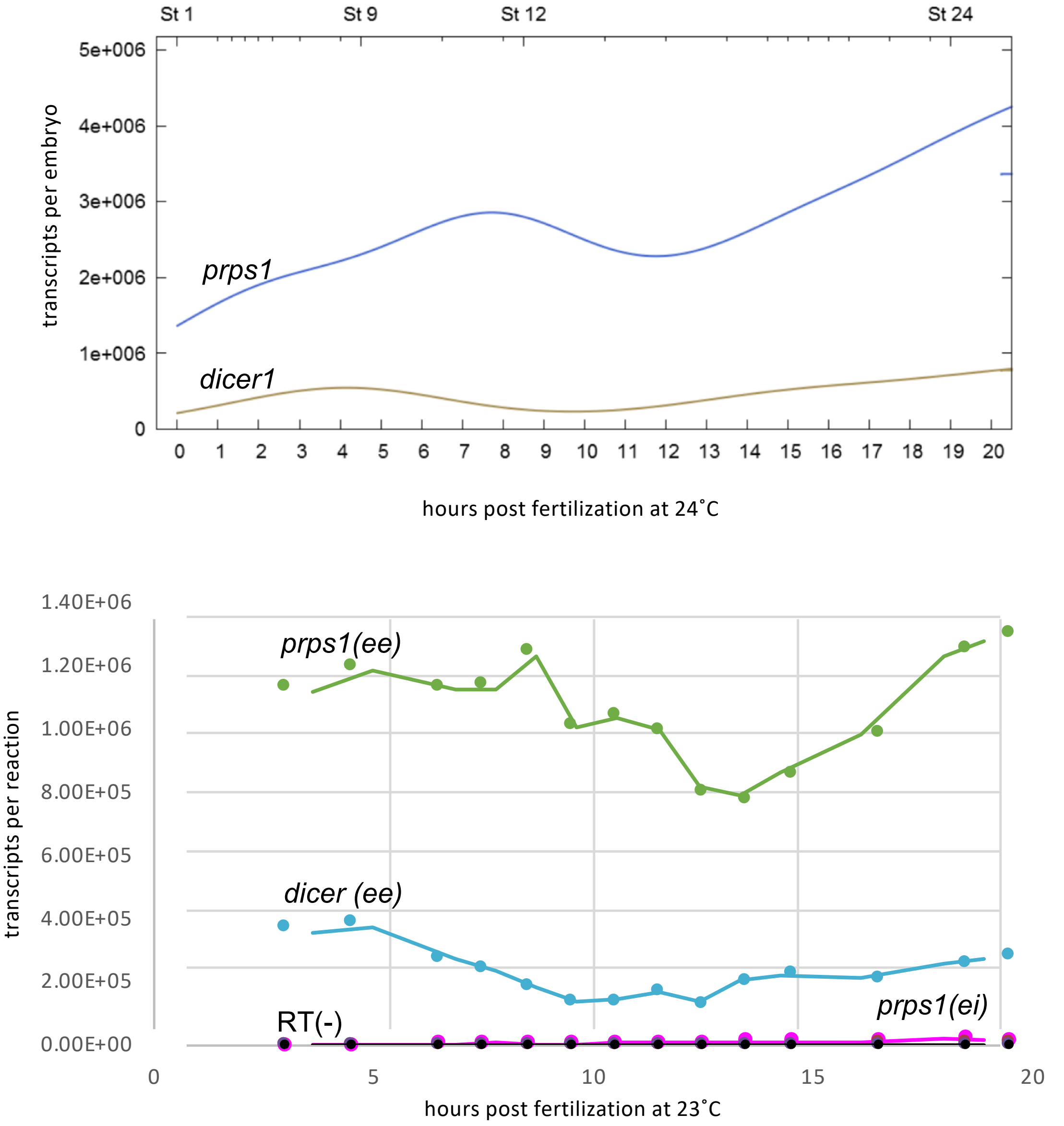

Fig. S2. Expression profiles of two housekeeping genes, *prps1* and *dicer1*.

Expression profiles of two housekeeping genes, *prps1* and *dicer1*, by RNA-seq (A) (Owens et al., 2016) and by qPCR (B) are shown. ee, mature RNA. ei, precursor RNA. The RNA-seq data was retrieved from http://genomics.crick.ac.uk/apps/profiles/. qPCR for all primer sets were performed with RT- samples (represented in black) to detect traces of contaminated genomic DNA. The copy numbers of pre-spliced *prps1* (ei) (magenta) were also low, but reached to about 20000 transcripts per reaction at 18.5 hpf. The amount of total RNA extracted per embryo was not different among stages, and the same amount of RNA was used per qPCR reaction. Therefore, the expression profiles of *prps1* and *dicer1* from RNA-seq and qPCR (ee) are directly comparable.

Biology Open • Supplementary information

Fig. S3 (Kondo et al.)

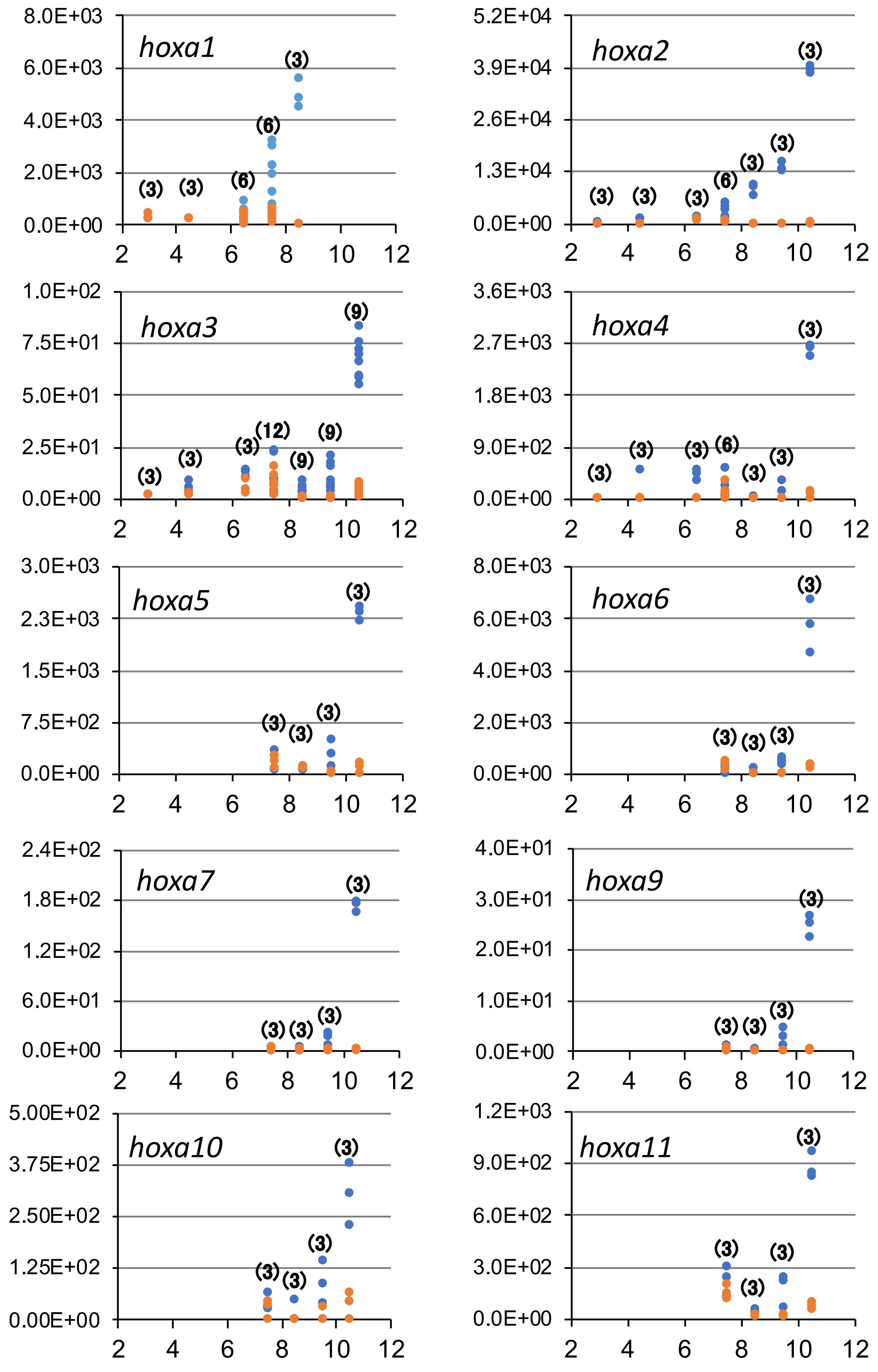

Fig. S3. Individual qPCR expression data of *hox* genes presented in Fig. 3.

The same data shown in Fig. 3 are replotted, in which each individual copy number per reaction is plotted as a dot for RT+ (blue) or RT- (orange) with the same number of replicates. The numbers in parentheses are sample sizes, between 3 and 12 replicates. Since the layer of blue dots is behind that of orange dots, some blue dots are hidden by orange dots. The x-axis represents hpf.

Fig. S3 (continued) (Kondo et al.)

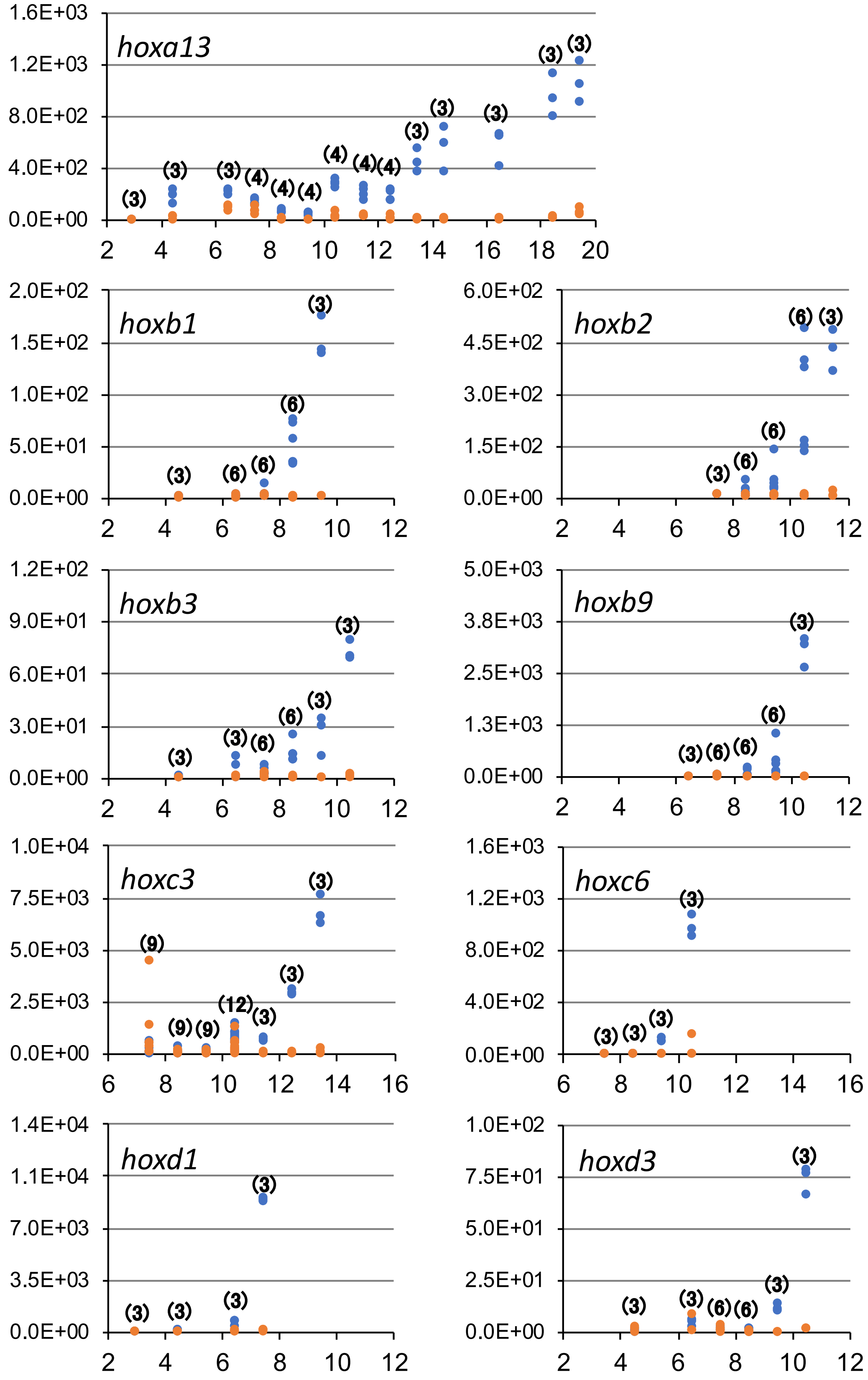

Fig. S4. The extrapolated onset method estimating *hox* gene activation.
Using the same data sets of qPCR as Fig. 3 or Fig. S3, the numbers of de novo synthesized transcripts were calculated. The data was analyzed using the Tukey-Kramer test and compared. Significant differences among data points are indicated in lowercase alphabets (for example, "b" represents significantly different from "a", while no significant difference was detected between the same alphabets). Two consecutive data points which show significant differences were connected and the intercept to the x-axis (time) was calculated as the onset of active transcription, except for *hoxc3*, where three points (10, 11.5, and 12.5 hpf) were used for the calculation (see Experimental Procedures).

Biology Open • Supplementary information

Fig. S4 (Kondo et al.)

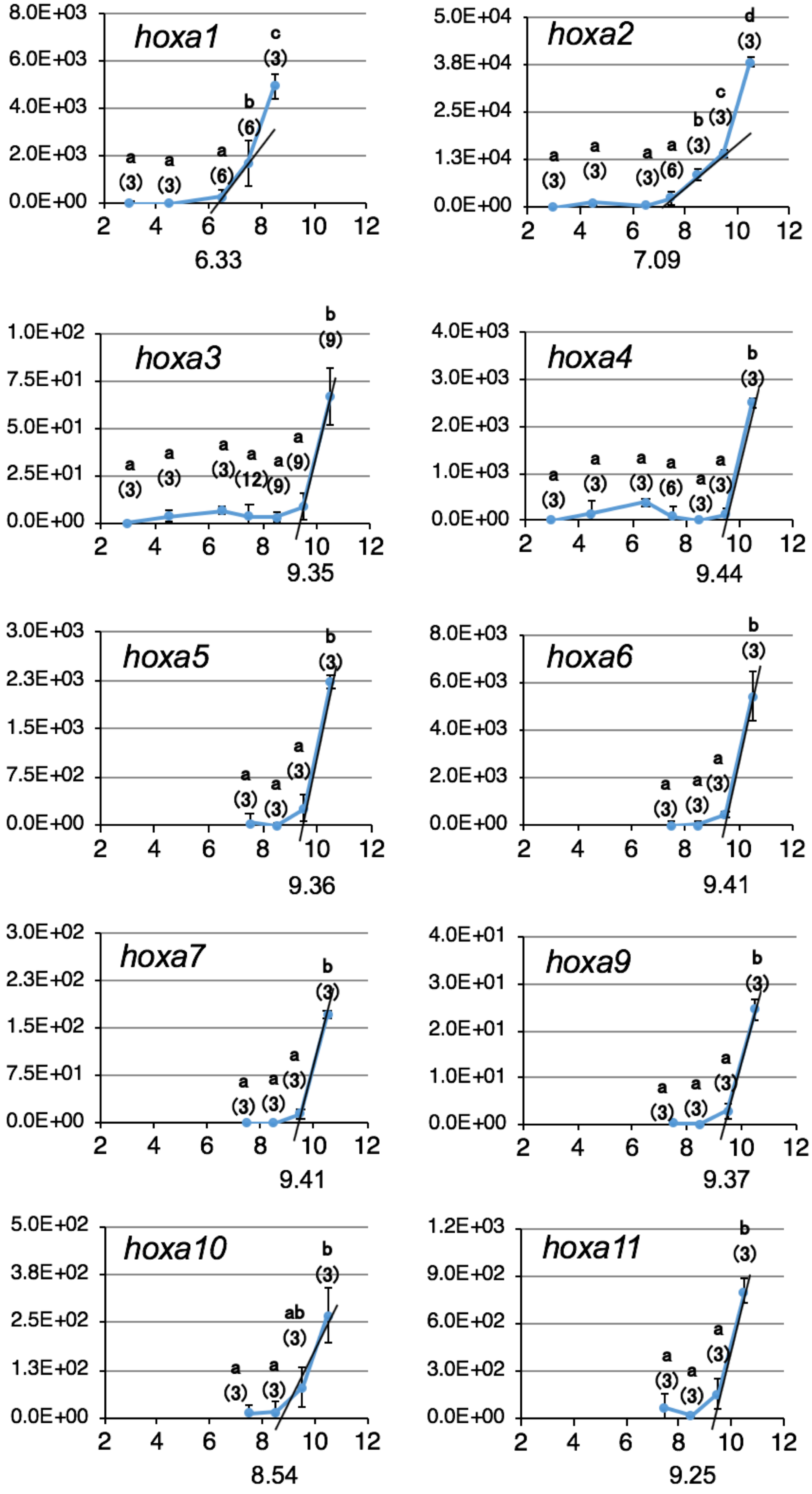

Biology Open • Supplementary information

Fig. S4 (continued) (Kondo et al.)

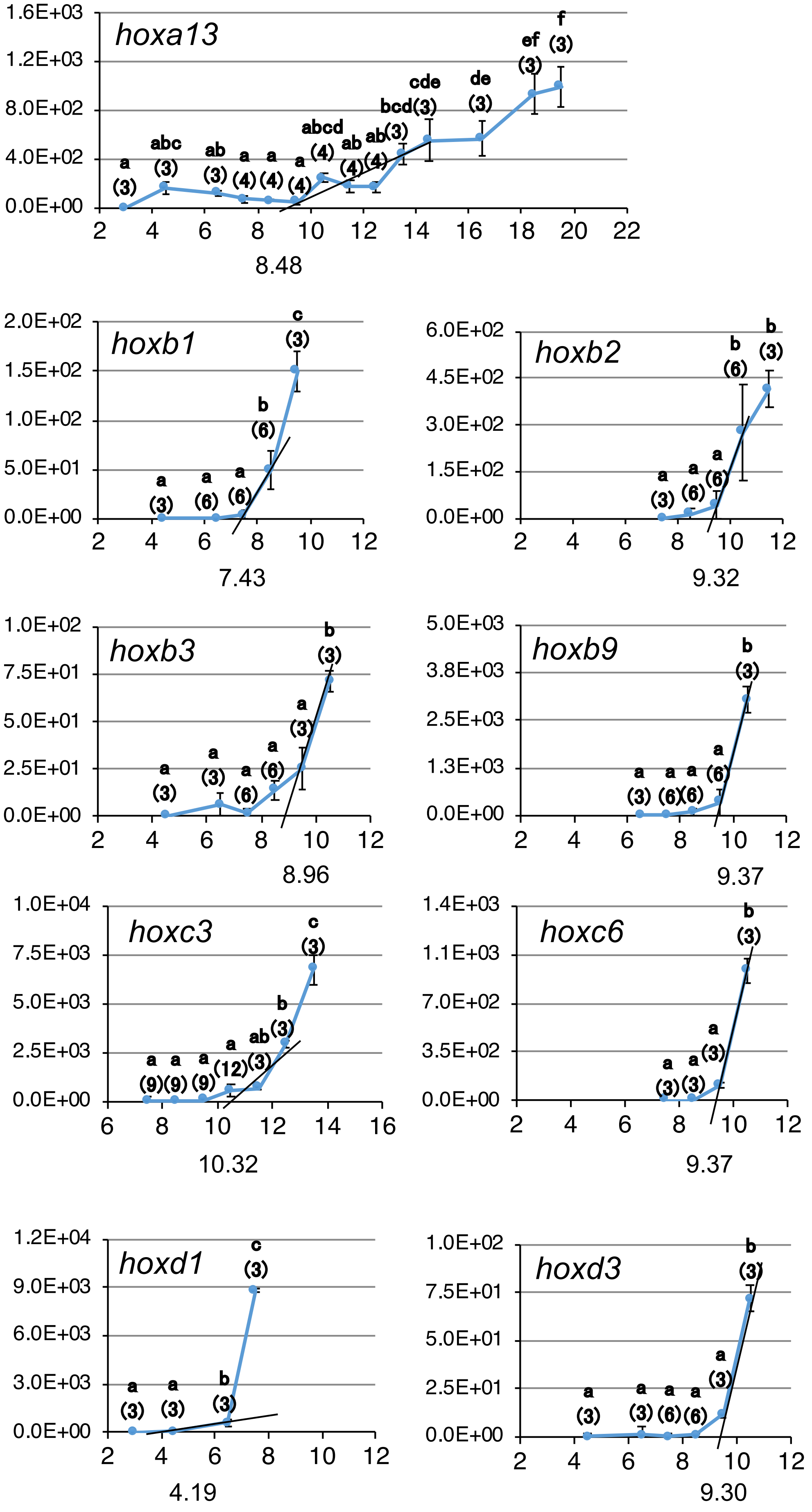

Biology Open • Supplementary information

Fig. S5 (Kondo et al.)

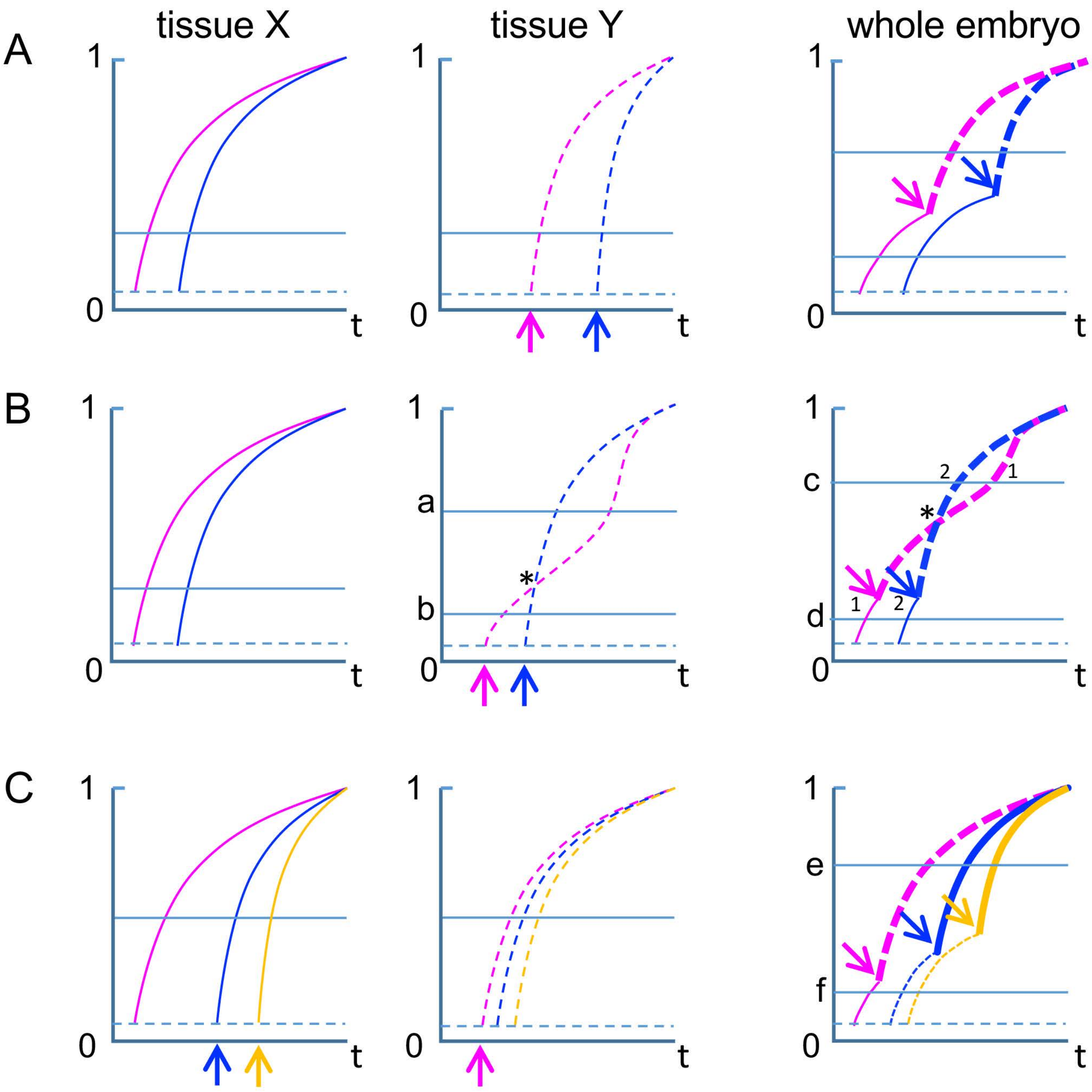

Fig. S5. Simulation of hypothetical genes expressed in the order of temporal collinearity.

Simulation of the time course of de novo expression of two or three *hox* genes *1* (magenta), *2* (blue), and *3* (yellow) in two tissues (X and Y, left and middle panels) and in the whole embryo (the sum of tissues X and Y, right panels) are depicted, assuming genes start expression in the order according to the temporal collinearity hypothesis, from gene *1* to *3* (anterior to posterior), and expression starts in tissue X earlier than tissue Y. x-axis, time (t); y-axis, the number of transcripts (normalized by the highest value as 1); vertical arrows in left and middle panels, the late expression in tissue X or Y compared to the other; oblique arrows in the right-most panel, the start point of cumulative expression (thick lines); solid horizontal lines, a detectable number of transcripts; dashed horizontal lines, detection limits; asterisks, the crossing point of transcript number between genes. (A) Simple monotonous increment of de novo transcripts in all genes. The anterior gene *1* always reaches a detectable number (horizontal lines) earlier than the posterior gene *2*. (B) Non-monotonous increment of de novo transcripts in tissue Y. In tissue Y, the order to reach a higher number (a) is reversed after the crossing point (asterisks) than a low number (b). This is the same in a whole embryo (c vs d). (C) Different timing of initial transcription of three genes in tissues X and Y. The three genes in tissue Y start to be transcribed between the start of expression of gene *1* and *2* in tissue X. In the whole embryo, the order that gene transcripts reach a high number (e) or a lower number (f) are the same, matching the order of genes.

Biology Open • Supplementary information

Table S1: List of primers used in the study

gene	primer name	sequence	position	annealing temperature
dicer	Xtdicer1-f	TGCTGAGAAAACCCTTGACCA	exon	55
	Xtdicer1-r	TGGTAAGAGGCATGTGTAAAAGC	exon	
prps1	Xtprps1-f1	TGACATGGCAGATACGTGTG	exon	60
	Xtprps1-r1	CAGGGCCCGAGAATATACC	exon	
	Xtprps1-r2i	AATCTGGGCACCAGCATTAC	intron	60 (with f1)
hoxa1	Xthoxa1-f1	TCTCCTTCCAGCGAAACATC	exon	60
	Xthoxa1-r1	ACAAGGGCGCCTTAATAGAG	intron	
hoxa2	Xthoxa2-f1	ACGGCACAATGGAGTTTACTG	intron	60
	Xthoxa2-r1	CCGGGAGAAGGCAGAACTAAG	intron	
hoxa3	Xthoxa3-f1	CGATGGCGCCTACATGTGTA	intron	60
	Xthoxa3-r1	TTGGAACCTGGGCTTCTTGG	intron	
hoxa4	Xthoxa4-f1	GCCAGAGGATTTATTGGAGTCC	intron	60
	Xthoxa4-r1	AAGGCTTCAGAGGAGCATGG	intron	
hoxa5	Xthoxa5-f1	ATTGGGTCGGTCAGATGAGG	intron	60
	Xthoxa5-r1	CTACACTGGGCTCTCACTGG	intron	
hoxa6	Xthoxa6-f1	CCCTGTCTATCCCTGGATGC	exon	60
	Xthoxa6-r1	GGTCCCTGTTCCACTTTGTC	intron	
hoxa7	Xthoxa7-f1	ACATCAACAAAGGGGTGAGCT	intron	55
	Xthoxa7-r1	GGTCATAAGCATTCCCTTCCCT	intron	
hoxa9	Xthoxa9-f1	GCTCAATGGCAGGGAGAGAA	intron	55
	Xthoxa9-r1	GTTCCCTTTGCGACTGAAGC	intron	
hoxa10	Xthoxa10-f1	AACTTCACACCACATGCCTG	intron	60
	Xthoxa10-r1	GGCTATGAGTGCACCCTTTG	intron	
hoxa11	Xthoxa11-f1	AGACAAAGAAGCCCTGCGTG	intron	60
	Xthoxa11-r1	CTGCTGTAAAACGTGTCCCC	intron	
hoxa13	Xthoxa13-f2	GATTGCGGCTGAAGTTGGAG	intron	60
	Xthoxa13-r3	GCACGACATCTGCAAAGGAC	exon/intron junction	
hoxb1	Xthoxb1-f1	GCCTCCGCTTCACCTTATATCC	intron	60
	Xthoxb1-r1	TTTATTGCAGGGGTGGGAGC	intron	
hoxb2	Xthoxb2-f1	CTGGGCCATAACAGTGACGA	intron	60
	Xthoxb2-r1	CCATGTGGCCAGTGGATTTC	intron	
hoxb3	Xthoxb3-f1	TGTATAGTCCAGTCGCTGTAGG	intron	60
	Xthoxb3-r1	TCAAGCGATGCAGACAGTTG	intron	
hoxb9	Xthoxb9-f1	CCACTAAACTGGGCACGGAT	intron	55
	Xthoxb9-r1	CACAGCGTTTTGTCAGCCTG	intron	
hoxc3	Xthoxc3-f2	CCTTGGATGAAGGAAACTCG	exon	60
	Xthoxc3-r2	TCCCCATCAACAGGTAAAGC	intron	
hoxc6	Xthoxc6-f1	TTCATGGTTGCACGGGTTTG	intron	55
	Xthoxc6-r1	CCCTGCCCAGAAACGACTTT	intron	
hoxd1	Xthoxd1-f2	CAGTAGCCAGAATATCCATGCG	intron	60
	Xthoxd1-r2b	TGCTACCCCATATTCAGATTGTAAAC	exon	
hoxd3	Xthoxd3-f2	GAACAGAGAGGGTGAGTGGG	intron	60
	Xthoxd3-r2	AGCCATTGAGCCATTGAGCC	intron	

Biology Open • Supplementary information
